# Transcriptional Immunoprofiling at the Tick-Virus-Host Interface during Early Stages of Tick-Borne Encephalitis Virus Transmission

**DOI:** 10.3389/fcimb.2017.00494

**Published:** 2017-12-01

**Authors:** Saravanan Thangamani, Meghan E. Hermance, Rodrigo I. Santos, Mirko Slovak, Dar Heinze, Steven G. Widen, Maria Kazimirova

**Affiliations:** ^1^Department of Pathology, The University of Texas Medical Branch, Galveston, TX, United States; ^2^Institute for Human Infections and Immunity, The University of Texas Medical Branch, Galveston, TX, United States; ^3^Center for Tropical Diseases, The University of Texas Medical Branch, Galveston, TX, United States; ^4^Institute of Zoology, Slovak Academy of Sciences, Bratislava, Slovakia; ^5^Department of Surgery, Center for Regenerative Medicine, Boston University and Boston Medical Center, Boston, MA, United States; ^6^Department of Biochemistry and Molecular Biology, The University of Texas Medical Branch, Galveston, TX, United States

**Keywords:** TBEV, flavivirus, tick, *Ixodes ricinus*, cutaneous, immune response

## Abstract

Emerging and re-emerging diseases transmitted by blood feeding arthropods are significant global public health problems. Ticks transmit the greatest variety of pathogenic microorganisms of any blood feeding arthropod. Infectious agents transmitted by ticks are delivered to the vertebrate host together with saliva at the bite site. Tick salivary glands produce complex cocktails of bioactive molecules that facilitate blood feeding and pathogen transmission by modulating host hemostasis, pain/itch responses, wound healing, and both innate and adaptive immunity. In this study, we utilized Illumina Next Generation Sequencing to characterize the transcriptional immunoprofile of cutaneous immune responses to *Ixodes ricinus* transmitted tick-borne encephalitis virus (TBEV). A comparative immune gene expression analysis of TBEV-infected and uninfected tick feeding sites was performed. Our analysis reveals that ticks create an inflammatory environment at the bite site during the first 3 h of feeding, and significant differences in host responses were observed between TBEV-infected and uninfected tick feeding. Gene-expression analysis reveals modulation of inflammatory genes after 1 and 3 h of TBEV-infected tick feeding. Transcriptional levels of genes specific to chemokines and cytokines indicated a neutrophil-dominated immune response. Immunohistochemistry of the tick feeding site revealed that mononuclear phagocytes and fibroblasts are the primary target cells for TBEV infection and did not detect TBEV antigens in neutrophils. Together, the transcriptional and immunohistochemistry results suggest that early cutaneous host responses to TBEV-infected tick feeding are more inflammatory than expected and highlight the importance of inflammatory chemokine and cytokine pathways in tick-borne flavivirus transmission.

## Introduction

Tick-borne encephalitis virus (TBEV) is a zoonotic tick-borne virus in the *Flaviviridae* family (genus *Flavivirus*). It is the causative agent of tick-borne encephalitis (TBE), a serious neurological disease in humans. During the last few decades, TBE has become a widespread public health concern in Eurasia with endemic regions extending from Western and Central Europe to Siberia and parts of Asia (Süss, 2011). The various strains of TBEV are subdivided into three main subtypes that are closely related genetically and antigenically: European (Eu), Siberian (Sib), and Far-Eastern (FE) (Gritsun et al., 2003a; Mansfield et al., 2009). TBEV-Eu is widely distributed in Europe, including the European regions of Russia, while TBEV-Sib is mainly found in Russia, the Baltic countries and Finland (Mansfield et al., 2009; Kovalev and Mukhacheva, 2014). TBEV-FE is present in Far-Eastern Russia and parts of China, Japan, and the Republic of Korea (Mansfield et al., 2009). Human infections with TBEV can range from mild flu-like symptoms to severe or fatal neuroinvasive disease, often with long-term neurological symptoms. There is a correlation between the TBEV subtype and severity of disease. TBEV-FE is associated with severe neurological disease and a case fatality rate of approximately 30–40%, while the case fatality rates for TBEV-Sib and TBEV-Eu are approximately 6–8% and 1–2%, respectively (Gritsun et al., 2003a; Tonteri et al., 2013). Although the incidence rates vary from year to year and between subtypes, several thousand human TBE cases are reported annually (CDC Tick-borne Encephalitis, 2017).

In nature, the *Ixodes ricinus* tick is the primary vector for TBEV-Eu while the *Ixodes persulcatus* tick is the main vector for the TBEV-Sib and TBEV-FE (Gritsun et al., 2003b). *I. ricinus* is widely distributed throughout Europe, extending to Turkey, and northern Iran, while *I. persulcatus* is distributed across the Urals, Siberia, Far-Eastern Russia, and parts of China and Japan (Gritsun et al., 2003a; Lindquist and Vapalahti, 2008). A sympatric zone exists in northern Baltics, western Finland and northwestern Russia where the habitats for *I. ricinus* and *I. persulcatus* overlap and multiple TBEV subtypes have been recorded (Lindquist and Vapalahti, 2008; Süss, 2011; Kovalev and Mukhacheva, 2014). TBEV is maintained in natural transmission cycles involving ixodid ticks and wild-living mammalian hosts. When infected with TBEV, a tick is supposed to remain infected throughout its life cycle (Gritsun et al., 2003a). Transovarial transmission of TBEV from an infected female tick to the egg mass can occur, but this route of tick infection is not entirely efficient at maintaining TBEV within the natural tick population (Danielová et al., 2002). During the tick feeding process, TBEV-infected ticks can transmit the virus to susceptible vertebrate hosts, but they can also transmit TBEV to uninfected ticks that are co-feeding on the same host (Mansfield et al., 2009; Randolph, 2011). During co-feeding, TBEV can be transmitted even non-viremically i.e., when the ticks feed on a non-viremic or virus- immune host (Labuda et al., 1993, 1997). The local skin site of tick feeding is understood to be an important focus for early TBEV replication, and immune cell infiltrates to this feeding site are believed to serve as vehicles for TBEV transmission between co-feeding ticks (Labuda et al., 1996).

Infectious agents transmitted by ticks are delivered to the vertebrate host together with saliva at the tick feeding site. Tick-borne viruses are transmitted to the host very early during the tick feeding process. TBEV can be transmitted from the saliva of an *I. ricinus* tick to the cement cone in the skin of a host as early as 1 h after the tick attaches and initiates feeding (Alekseev et al., 1996). As *I. ricinus* and *I. persulcatus* ticks feed, TBEV replicates to higher viral titers than in unfed ticks (Alekseev and Chunikhin, 1990; Belova et al., 2012; Slovák et al., 2014). The dynamic nature of TBEV replication in ticks has also been demonstrated in field-collected ticks. In partially engorged *I. ricinus* nymphs removed from humans, TBEV prevalence was higher than in questing, unfed nymphs collected in the same region (Süss et al., 2004). Experimental data suggest that in nature, ticks secrete repeated “pulses” of a few infectious virus particles over the course of feeding (Kaufman and Nuttall, 1996). Thus, virus transmission from an infected tick to a host is a very dynamic process that begins soon after the tick initiates feeding.

Ixodid ticks must remain attached to their hosts for several days to successfully acquire a bloodmeal and complete development, and have evolved salivary countermeasures directed against the host's immune and hemostatic defenses. Tick salivary glands produce complex cocktails of biologically active molecules that facilitate blood feeding and pathogen transmission by modulating host hemostasis, pain/itch responses, wound healing, and both innate and adaptive immunity. Bioactive tick salivary molecules include those with anti-pain/itch, antiplatelet, anticoagulation, vasodilatory, immunomodulatory, and anti-inflammatory activities (Ribeiro et al., 2006, Francischetti et al., 2009; Kazimírová and Štibrániová, 2013; Wikel, 2013; Šimo et al., 2017). As the course of tick feeding progresses, salivary gland genes are differentially expressed, reflecting the dynamic and complex composition of tick saliva (Ribeiro et al., 2006; Šimo et al., 2017).

The skin is the first host organ that tick saliva and a tick-borne pathogen contact during the tick feeding process. The cutaneous interface between the tick, pathogen, and host is crucial for influencing the initial host response to tick infestation and pathogen transmission (Kazimírová et al., 2017; Šimo et al., 2017). A prior study examined the tick-induced changes in cutaneous gene expression and histopathology during the early stages of uninfected *Ixodes scapularis* feeding. Early transcriptional and histopathological changes at the feeding site of uninfected *I. scapularis* nymphs are initially characterized by modulation of host responses in resident cells, followed by progression to a neutrophil-dominated immune response after 12 h of tick feeding (Heinze et al., 2012). Similarly, a complex proinflammatory environment was observed at the Powassan virus (POWV), a North American tick-borne flavivirus, infected tick feeding site. Together these findings from the cutaneous interface provide evidence of an immunologically privileged micro-environment at the tick feeding site that is established during the early stages of POWV-infected tick feeding (Hermance and Thangamani, 2014; Hermance et al., 2016).

In the present study, Illumina Next Generation Sequencing (NGS) and immunohistochemistry are utilized to understand host immunomodulation induced by TBEV-infected *I. ricinus* feeding at the earliest stages of TBEV transmission. By studying the interactions between the host immune response and tick-mediated immunomodulation during the early hours of infected tick feeding, we can begin to understand the immunologic processes that facilitate transmission of a tick-borne flavivirus to a host.

## Materials and methods

### Ethics statement

All experiments involving mice were performed in accordance with the animal use protocol approved by the State Veterinary and Food Administration of the Slovak Republic (permit number 1335/12-221).

### Animals

Five-week-old female BALB/c mice were purchased from Dobra Voda Breeding Station, Institute of Experimental Pharmacology and Toxicology, Slovak Academy of Sciences (SAS). The animals were housed at the Institute of Virology, Biomedical Research Center, SAS (Bratislava, Slovakia) under standard conditions. Food and water were provided *ad libitum*. Upon arrival, mice were allowed to adapt to the local environment before being incorporated into the study. Mice were 6 weeks old at the start of the study. At the end of the experiment animals were euthanized by cervical dislocation under anesthesia induced by carbon dioxide.

### Virus and tick infection

*I. ricinus* ticks were obtained from a laboratory colony maintained at the Institute of Zoology SAS (Bratislava, Slovakia). The F1 generation of laboratory-bred *I. ricinus* females was used for virus inoculation. TBEV (Hypr strain prepared as a 10% mouse brain suspension of 1.1 × 10^9^ PFU/ml in Leibovitz's L-15 medium) was provided by the Institute of Virology, Biomedical Research Center, SAS. Fasting *I. ricinus* females were inoculated into the haemocoel with TBEV (5.5 x 10^4^ PFU per tick) through the coxal plate of the second pair of legs using a digital microinjector TM system (MINJ-D-CE; Tritech Research, Inc., USA) (Kazimírová et al., 2012). By this procedure, ~100% of the ticks were found to acquire TBEV infection (Slovák et al., 2014). Inoculated ticks were incubated at room temperature and 85% relative humidity in a desiccator for 21 days prior to the infestation experiments.

### Infestation of mice by ticks

Two groups of mice (*n* = 6 per group) were infested with either TBEV-infected or uninfected (control) *I. ricinus* females. Ticks were placed in small neoprene capsules glued on the shaved backs of the mice (two capsules per mouse, four tick females per capsule) (Kazimírová et al., 2012; Hermance and Thangamani, 2014). Ticks in each capsule were allowed to feed for either 1 or 3 h. After the allotted feeding time, skin biopsies were taken from euthanized mice, at each tick feeding site using a Premier Uni-Punch (Premier Products Co., Plymouth Meeting, PA). For immunohistochemical analysis, skin biopsies (*n* = 3) were harvested with attached ticks and placed in 4% formaldehyde. For RNA extraction, the attached ticks were removed from the skin biopsies and the biopsies (*n* = 3) were placed in RNALater (Ambion, Life Technologies, Carlsbad, CA). Control biopsies were taken from the shaved skin of naïve tick-free mice and stored in either 4% formaldehyde or RNALater.

### Cutaneous immune response

Total RNA was extracted as previously described (Heinze et al., 2012; Hermance and Thangamani, 2014) and RNAseq analysis (Illumina deep sequencing / NGS) was performed on these samples. Briefly, 1 μg of total RNA from mouse skin biopsies (*n* = 3) was poly A+ selected and fragmented using divalent cations and heat (94°C, 8 min). Illumina TruSeq v2 sample preparation kits (Illumina Inc., San Diego, CA) were used for the RNA-Seq library construction. Each sample library was uniquely indexed to allow combining libraries during sequencing and subsequent separation post-sequencing. NGS was performed at the NGS core facility, Sealy Center for Molecular Medicine, The University of Texas Medical Branch (UTMB). Sample libraries were analyzed by the Illumina HiSeq 1500 using a 2 × 50 base paired end run protocol, with TruSeq v3 sequencing-by-synthesis chemistry. Reads were aligned to the mouse mm10 reference genome using TopHat version v2.0.4. Cuffdiff version 2.0.2 was used to estimate differential gene expression between TBEV-infected and uninfected feeding sites after 1 or 3 h of tick feeding. The total dataset of 23,000 genes was filtered for *p*-values ≤ 0.05 and a fold change ≤ −1.5 or ≥ +1.5. The Log_2_(fold change) and *p*-value data for each gene expression comparison (Supplement Table 1) were then uploaded to Ingenuity Pathway Analysis (IPA) software (Ingenuity Systems, Redwood City, CA) for further transcriptional analysis of the early cutaneous immune response, as previously described (Heinze et al., 2012; Hermance and Thangamani, 2014).

### Real-time PCR validation

Real-time PCR was used to validate the NGS data (Supplement Table 1). Skin biopsies were harvested from the feeding sites of TBEV-infected and uninfected ticks as described above. At each time point, biopsies from three mice were used for the real-time PCR validation. Fifteen gene targets were selected for real-time PCR analysis. We selected these genes based our previous studies with POWV where these genes were shown to be modulated genes of interest during the host anti-tick response to POWV infection (Hermance and Thangamani, 2014, 2015). This list also includes genes that were not differentially modulated as per RNASeq analysis. Primers were purchased from Integrated DNA Technologies and the primer sequences are provided in Supplement Table 2. Primers were mixed with IQ SYBR green supermix (Bio-Rad) and loaded into iCycler IQ PCR 96-well plates (Bio-Rad) to create customized PCR arrays where each gene was measured in triplicate. For each PCR plate, 1 μg of total RNA extracted from skin biopsies was converted into cDNA using the RT^2^ First Strand kit (Qiagen). cDNA was loaded onto the 96-well PCR plates, which were run on an iCycler iQ5 real-time PCR instrument (Bio-Rad) with the following cycling protocol: 10 min at 95°C; 15 s at 95°C, 1 min 60°C for 40 cycles, and an 80-cycle (+0.5°C /cycle) 55–95°C melt curve. Every array included GAPDH as an endogenous control gene and a no-template control. The iCycler's software was used to calculate the threshold cycle (C_T_) values for all analyzed genes. The delta-delta C_T_ method was used to calculate fold-changes in gene expression between TBEV-infected and uninfected tick feeding sites. Data normalization was achieved by correcting all C_T_ values to the average C_T_ values of the GAPDH housekeeping gene. Statistically significant differences in gene expression between the 1 vs. 3 h tick feeding time points were determined by the Student's *t*-test. *P*-values less than 0.05 were considered significant. SPSS statistical software was used.

### Immunohistochemistry

The skin biopsies with attached ticks were formalin-fixed for a minimum of 48 h in 4% formaldehyde. These biopsy samples were treated with Decal (Decal Chemical Corp, Tallman, NY) for 2 h, and then paraffin-embedded (Heinze et al., 2012). Five micron paraffin sections were taken from each sample and adhered to glass slides. The slides were deparaffinized in xylene and then rehydrated in decreasing concentrations of ethanol (Hermance et al., 2016). For antigen retrieval, the slides were treated with 10 mM Tris Base + 0.05% Tween 20 (pH 10) for 20 min with microwave heating. Upon returning to room temperature, endogenous peroxidase quenching was performed by incubating the slides in 4% H_2_O_2_ for 30 min. The primary antibody for TBEV detection used in this study was a Hyper Mouse Immune Ascitic Fluid (HMIAF) antibody against TBEV; therefore, the Mouse-On-Mouse kit (MOM, Vector labs, Burlingame, CA) was used in order to reduce the endogenous mouse Ig background staining generated when mouse primary antibodies are used on mouse tissue (Santos et al., 2016). Slides were incubated for 1 h at room temperature with the MOM mouse Ig blocking reagent. The HMIAF primary antibody against TBEV was diluted 1:250 in the MOM diluent and incubated for 30 min at room temperature. Secondary antibody consisted of MOM biotinylated horse anti-mouse IgG reagent, which was incubated for 30 min at room temperature. The biotinylated secondary antibody was detected with a streptavidin-peroxidase ultrasensitive polymer (Sigma-Aldrich, St. Louis, MO) followed by staining with the NovaRED HRP substrate kit (Vector Laboratories Inc., Burlingame, CA). Slides were counterstained with Harris hematoxylin and coverslips were mounted using Permount (ThermoFisher Scientific, Waltham, MA). Uninfected skin and tick biopsy sections generated from uninfected tick feeding sites were used as negative controls to verify the specificity of the MIAF anti-TBEV primary antibody. Secondary antibody only (no primary antibody) controls were used to confirm that the MOM biotinylated anti-mouse IgG reagent did not bind non-specifically to cellular components.

## Results and discussion

**Figure 1:**
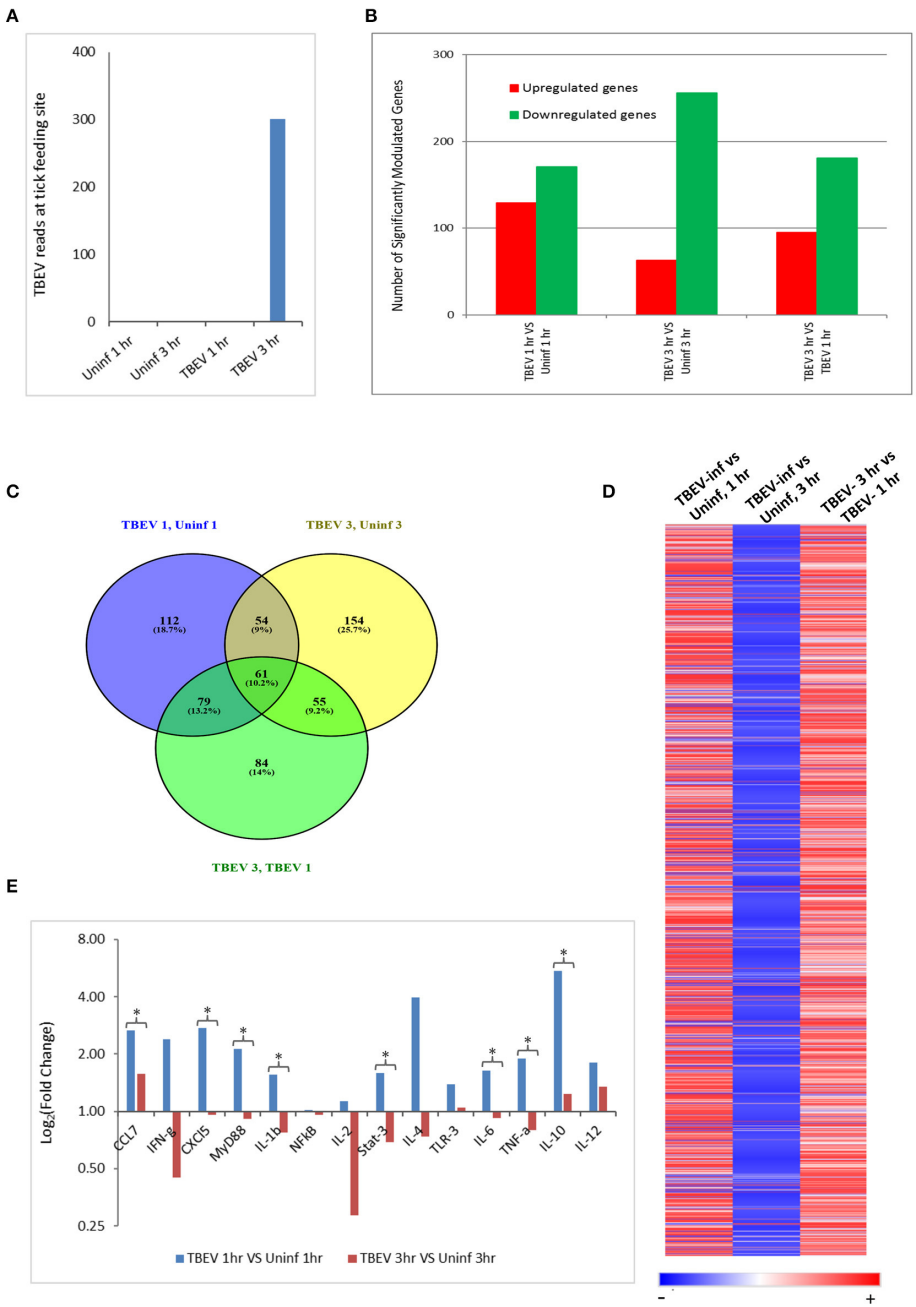
Comparative transcriptional analysis of the TBEV-infected and uninfected *Ixodes ricinus* tick feeding loci. **(A)** The RNASeq data was screened for the presence of TBEV reads at the tick feeding site by aligning the sequences against a TBEV reference genome (MN_001672). The number of TBEV reads that match the TBEV reference genome is plotted for the uninfected tick feeding sites and the TBEV-infected tick feeding sites. **(B,C)** The following comparisons are depicted: TBEV-infected tick feeding loci at 1 h vs. uninfected tick feeding loci at 1 h, TBEV-infected tick feeding loci at 3 h vs. uninfected tick feeding loci at 3 h, TBEV-infected tick feeding loci at 3 h vs. TBEV-infected tick feeding loci at 1 h. **(B)** The total number of significantly up- or downregulated (*p* ≤ 0.05) genes for each comparison. **(C)** Venn diagram showing overlap of significantly modulated genes for each of the three comparisons. **(D)** Heat map showing temporal changes in gene expression profiles. A list of all genes modulated at any time point in the study was used to generate a heatmap with Morpheus web server application (www.broadinstitute.org). **(E)** The immune genes selected for validation were shown to be modulated genes of interest during the host anti-tick response in previous studies. Pre-optimized primers were purchased from IDT and used for the real-time PCR validation. The delta-delta CT method was used to calculate fold-changes in gene expression between TBEV-infected and uninfected tick feeding sites as described in the methods section. GAPDH was used as an endogenous control gene. Statistically significant differences in gene expression between the 1 vs. 3 h tick feeding time points were determined by the Student's *t*-test. *P*-values less than 0.05 were considered significant. Significant differences are indicated by asterisks.

In the present study, the host cutaneous immune response to TBEV-infected *I. ricinus* feeding after 1 and 3 h of tick attachment was investigated by Illumina NGS and immunohistochemistry. No prior studies have examined the early host immune response at the feeding site of a TBEV-infected tick. We also used the RNASeq data to check for the presence of TBEV at the infected tick feeding site by aligning the sequences against a TBEV reference genome (NM_001672). TBEV sequences were detected in the 3 hpi (hours post infection), but not in the 1 hpi samples (Figure 1A).

The differences in the total number of significantly up- and downregulated host genes (*p* ≤ 0.05) between TBEV-infected and uninfected tick feeding sites as well as between TBEV-infected feeding sites 1 and 3 h post tick attachment are shown in Figure 1B. When the TBEV-infected and uninfected tick feeding sites were compared, the total number of significantly upregulated genes decreased from 1 to 3 h, while there was an overall increase in significantly downregulated genes from 1 to 3 h tick attachment. An online tool (Oliveros, 2007) was used to generate a Venn diagram showing the overlap of significantly modulated genes between the 1 and 3 h comparison of the TBEV-infected vs. uninfected tick feeding sites (Figure 1C). 10.2% of significantly modulated genes were shared between the three comparisons; however, the majority of significantly modulated genes were unique to either the 1 h TBEV-infected vs. 1 h uninfected tick feeding site (18.7%), the 3 h TBEV-infected vs. 3 h uninfected tick feeding site (25.7%), or the 3 h TBEV-infected vs. 1 h TBEV-infected tick feeding site (14%). Additionally, a list of all modulated genes at either time point in the study was used to generate a heat map (Figure 1D). This heat map suggests that after 1 h of TBEV-infected tick feeding, there was a pattern of mostly upregulated cutaneous genes; however, after 3 h, the pattern changed to downregulation. This pattern of gene expression was further validated by the real-time PCR data. Though we could not recapitulate the exact fold changes of the selected immune genes observed by RNASeq data, our data clearly concur with the expression pattern of the selected genes: upregulation at 1 hpi and downregulation at 3 hpi (Figure 1E). Together these data suggest that a distinctive cutaneous immune response profile exists after 1 h of TBEV-infected tick feeding, but it changes to reflect a new and unique profile after 3 h. This change in gene expression profile could be attributed to the dynamic salivary secretion and physical injury during tick attachment/feeding mechanisms.

### Ingenuity pathway analysis

**Figure 2:**
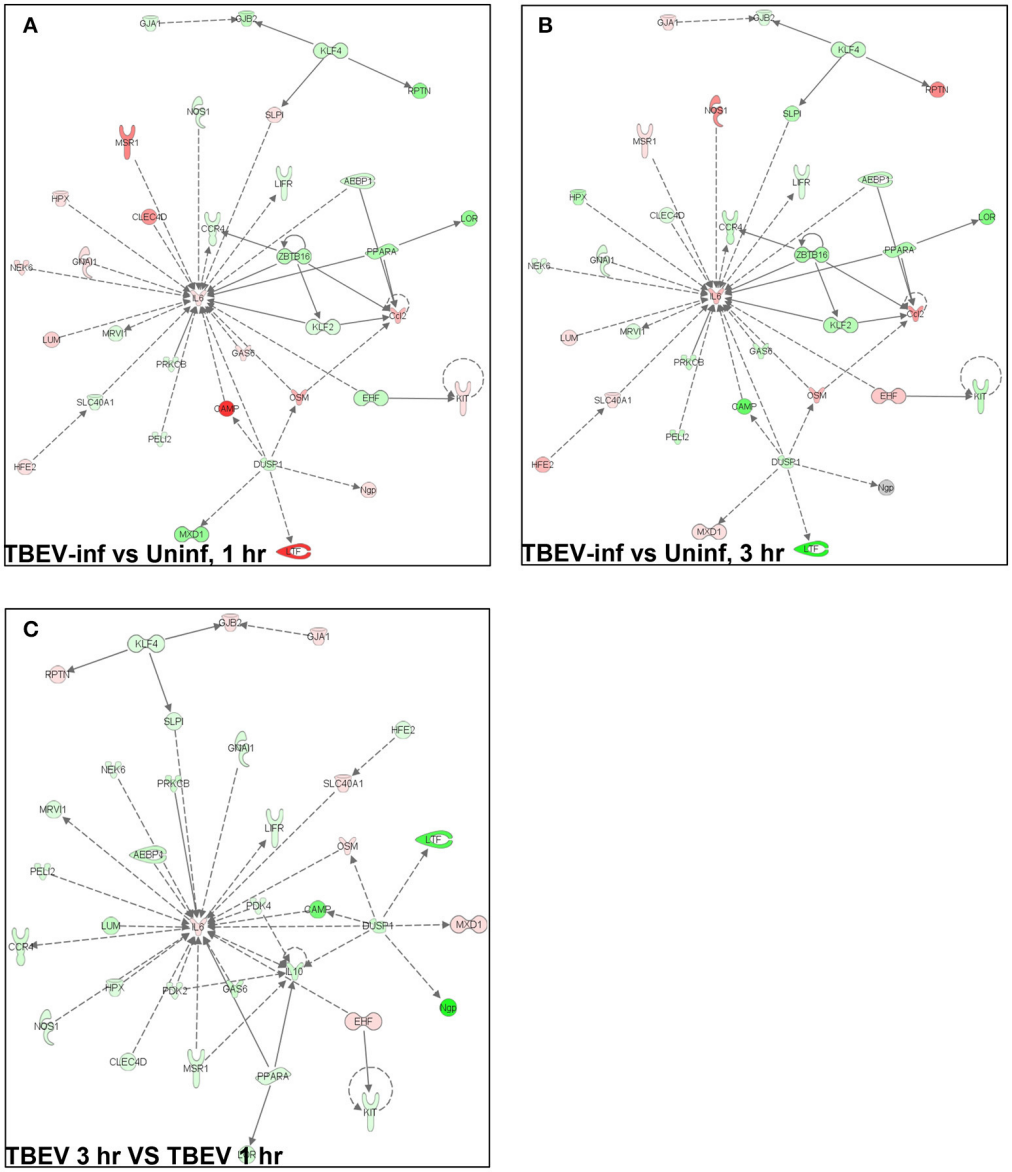
An overview of host gene modulation after 1 and 3 h of TBEV-infected *Ixodes ricinus* tick feeding. **(A)** The top IPA-generated network is shown for the TBEV-infected tick feeding loci at 1 h vs. the uninfected tick feeding loci at 1 h **(B)** The top IPA-generated network is shown for the TBEV-infected tick feeding loci at 3 h vs. the uninfected tick feeding loci at 3 h. **(C)** The top-IPA-generated network is shown for the TBEV-infected tick feeding loci at 3 h vs. TBEV-infected tick feeding loci at 1 h. Note: Red/pink represents upregulated genes, green represents down-regulated genes, and gray represents unchanged or insignificant genes.

In total, 1,548 genes were analyzed by Ingenuity Pathway Analysis (IPA) software (Supplement Table 1). The focus of the present study is on the cutaneous immune response observed at the TBEV-infected vs. the uninfected tick feeding sites after 1 and 3 h of tick feeding. The networks generated from IPA analysis illustrate the interrelationships between genes and the temporal changes in gene modulation. The top IPA-generated networks are shown for the TBEV-infected vs. uninfected tick feeding sites after 1 h of tick feeding (Figure 2A), the TBEV-infected vs. uninfected tick feeding sites after 3 h of tick feeding (Figure 2B), and the TBEV-infected tick feeding site after 3 h of tick feeding vs. the TBEV-infected tick feeding site after 1 h of feeding (Figure 2C). The three bio-functions most associated with the networks representing the TBEV-infected vs. uninfected comparisons (Figures 2A,B) are cellular movement, organismal injury and abnormalities, and hematological system development and function. After 1 h of TBEV-infected tick feeding, 19 host genes shown in the Figure 2A network were downregulated and 16 host genes were upregulated. After 3 h of TBEV-infected tick feeding, 22 host genes in the Figure 2B network were downregulated, 12 were upregulated, and one gene (Ngp) had a fold change of zero. Thus, as 1 h progressed to 3 h of TBEV-infected tick feeding, the network displayed a slight shift toward cutaneous gene downregulation (Figure 2). The IPA-generated network for the TBEV-infected 3 h vs. the TBEV-infected 1 h tick feeding site (Figure 2C) is unique from the Figures 2A,B networks, as it lacks Ccl2, KLF2, and ZBTB16, but instead includes 20 unique genes which contribute to this network's association with bio-functions such as molecular transport, organismal injury and abnormalities, and cellular movement.

**Figure 3:**
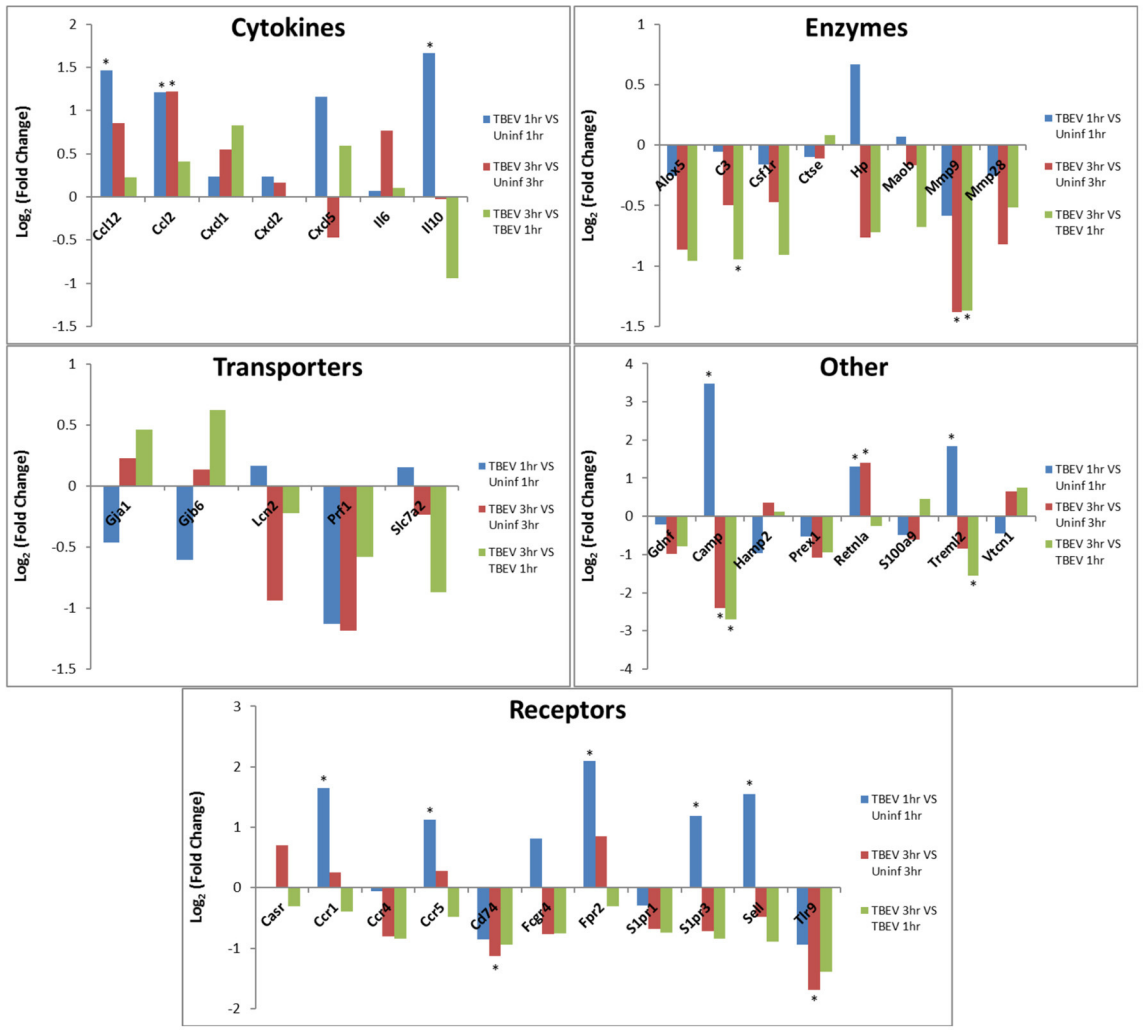
Changes in gene expression related to the inflammatory response after 1 and 3 h of TBEV-infected *Ixodes ricinus* tick feeding. Temporal changes in gene expression data were analyzed by IPA software. Genes predicted to be directly involved in the “Inflammatory Response” were plotted to show temporal changes in gene expression for the following comparisons: TBEV-infected tick feeding loci at 1 h vs. uninfected tick feeding loci at 1 h, TBEV-infected tick feeding loci at 3 h vs. uninfected tick feeding loci at 3 h, TBEV-infected tick feeding loci at 3 h vs. TBEV-infected tick feeding loci at 1 h. Genes with significant modulation in expression (*p* ≤ 0.05) are marked with an asterisk.

In both the 1 and 3 h comparisons between TBEV-infected vs. uninfected tick feeding sites, inflammatory response was the primary predicted host response. Based on the IPA transcriptional immunoprofiling, the inflammatory response was projected to be activated in the 1 h comparison of the TBEV-infected vs. uninfected tick feeding site (activation z-score = 1.549), and inhibited in the 3 h comparison (activation z-score = −1.26). Temporal changes in gene expression for all genes predicted to have direct correlative relationships with the inflammatory response were plotted after 1 or 3 h of TBEV-infected vs. uninfected tick feeding (Figure 3). Transcriptional levels of cytokines Ccl2, Ccl12, Cxcl1, Cxcl2, Cxcl5, IL6, and IL10 were all upregulated after 1 h of TBEV-infected tick feeding, thus contributing to the overall activation of the inflammatory response (Figure 3). At the 3 h time point, the majority of these cytokine transcriptional level were still upregulated, with the exception of Cxcl5 and IL10, which were both slightly downregulated. Real-time PCR validation of a few selected immune genes followed the general pattern observed in the Illumina NGS analysis (Figure 1E). All of the enzymes projected to have a direct, correlative relationship with the inflammatory response were downregulated at 3 h, further contributing to the predicted inhibition of the inflammatory response at the feeding site of a TBEV-infected tick (Figure 3). Additionally, transcript expression of several receptors (CCR1, CCR5, and Sell) were all significantly upregulated after 1 h of TBEV-infected tick feeding (Figure 3), likely contributing to the overall accumulation of immune cells to the tick attachment site. In summary, the cutaneous gene expression analysis indicates that the inflammatory response is activated after only 1 h of TBEV-infected tick feeding, and that increased recruitment and accumulation of immune cells is expected at the 1 h time point. However, after 3 h of TBEV-infected tick feeding, the cutaneous inflammatory response is predicted to undergo slight inhibition, as demonstrated by the downregulated expression of over half of the genes shown to have a correlative relationship with the inflammatory response (Figure 3).

**Figure 4:**
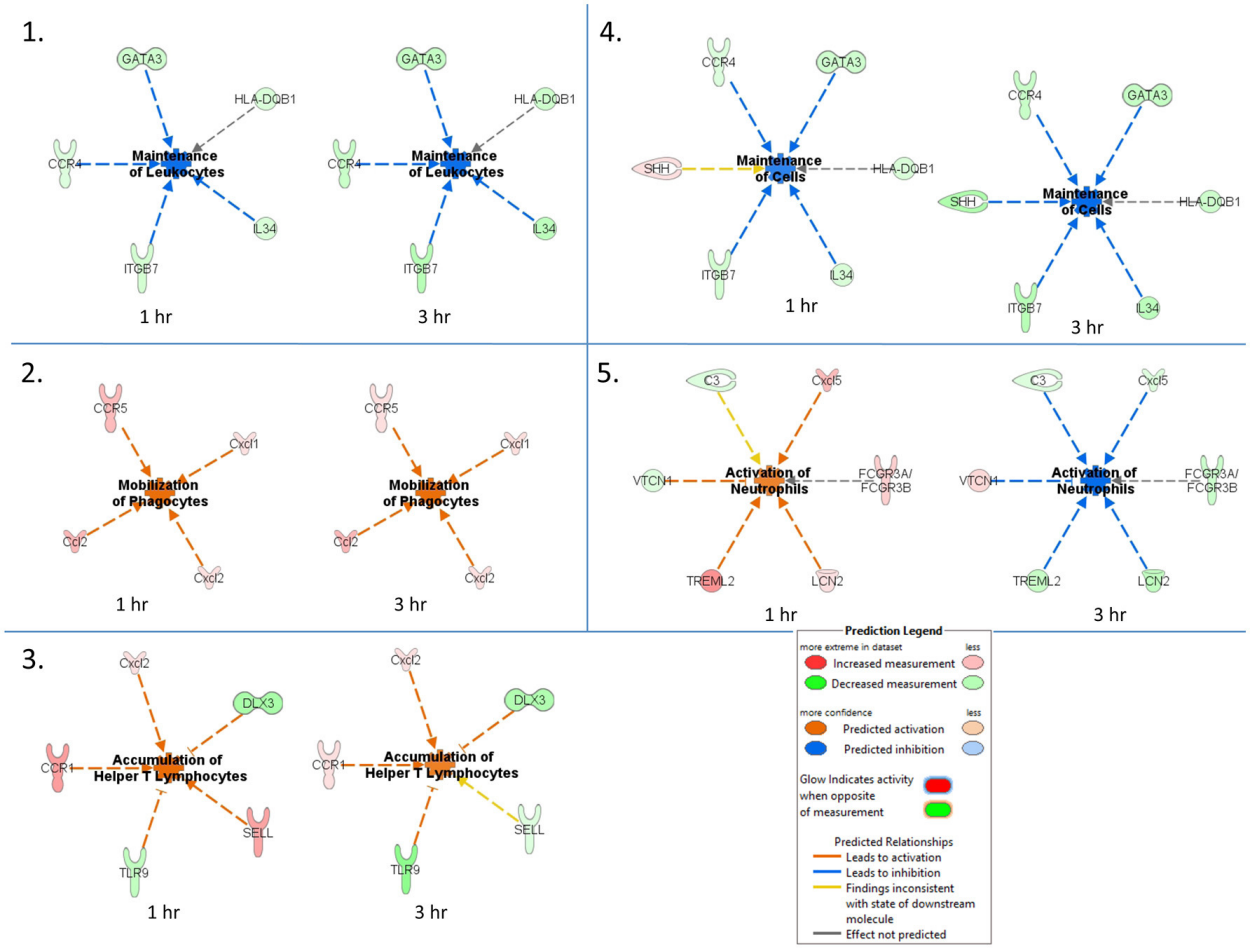
Activation status prediction networks for the top five immune responses / bio-functions generated from the IPA core comparison analysis. An IPA core comparison analysis was used to analyze which biological processes are relevant at 1 and 3 h of TBEV-infected vs. uninfected tick feeding. The predicted activation status of each network is shown.

An IPA core comparison analysis was used to analyze changes in host cutaneous gene expression in response to TBEV-infected tick feeding vs. uninfected tick feeding observed across the two experimental time points (1 and 3 h). Figure 4 shows activation status prediction networks for the top five immune responses or bio-functions generated from the IPA core comparison analysis. “Maintenance of leukocytes” was the top predicted bio-function in the core comparison analysis (Figure 4). This IPA category moniker refers to bio-functions associated with the normal cellular activities that maintain cellular homeostasis, including engulfment, phagocytosis, regulation, and stasis of cells. The maintenance of leukocytes was predicted to be inhibited at both the 1 and 3 h comparisons of TBEV-infected vs. uninfected tick feeding (activation z-score = −2 for both time points). At both the 1 and 3 h time points, GATA3 transcription was downregulated (Figure 4). GATA3 is a transcription factor required for both the maintenance and development of Th2 cells (Pai et al., 2004). Furthermore, IL34, which is highly expressed by keratinocytes in the epidermis and plays an important role in the maintenance and development of Langerhans cells (Greter et al., 2012), was also downregulated after 1 and 3 h of TBEV-infected tick feeding (Figure 4). CCR4 knockout (CCR4^−/−^) bone marrow-derived dendritic cells are less efficient in the maintenance of Th17 responses compared to wild type dendritic cells (Poppensieker et al., 2012); therefore, the downregulation of CCR4 transcription in the present study contributes to the inhibition of leukocyte maintenance at the 1 and 3 h time points. Mouse integrin subunit beta 7 (ITGB7) is necessary for the maintenance of memory T lymphocytes expressing the CD4 protein (Yang et al., 2011). Since ITGB7 is downregulated in the present transcriptional immunoprofiling study, it likely contributes to the inhibited maintenance of memory CD4+ T cells after 1 and 3 h of TBEV-infected tick feeding.

“Mobilization of phagocytes” was the second predicted bio-function in the core comparison analysis. After 1 and 3 h of TBEV-infected vs. uninfected tick feeding, the mobilization of phagocytes was predicted to be activated (activation z-score = 1.982 for both time points) (Figure 4). Monocytes, macrophages, neutrophils, dendritic cells, and mast cells are several of the professional phagocytic cells. In mice, there are two Ccl2 homologs, Ccl2 and Ccl12, both of which are potent phagocyte chemoattractants. The transcript levels of these two chemokines are upregulated at both the 1 and 3 h time points, thereby increasing the mobilization and chemotaxis of phagocytes at the TBEV-infected tick feeding site (Figures 3, 4). CCR5 is known to regulate accumulation of activated macrophages in the West Nile virus-infected mouse brain (Glass et al., 2005), and upregulated transcription of this chemokine receptor in the present study likely contributes to the activated mobilization of phagocytes. Mast cells and tissue resident macrophages release the potent neutrophil chemoattractants, Cxcl1 and Cxcl2 (De Filippo et al., 2008, 2013). In response to tissue inflammation, mast cells positioned near the vasculature initiate neutrophil infiltration from the circulation by releasing Cxcl1 and Cxcl2, while tissue resident macrophages also release Cxcl1 and Cxcl2, recruiting neutrophils deeper into the inflamed tissue (De Filippo et al., 2013). In the present study transcript levels of both these chemokines are both upregulated during TBEV-infected tick feeding, thus contributing to the activated mobilization of neutrophils that infiltrate the tick feeding site very early (Figures 3, 4).

The third predicted bio-function generated in the IPA core comparison analysis was “accumulation of helper T lymphocytes.” The activation z-score for this bio-function decreased from 2.19 at 1 h post-TBEV-infected tick feeding to 1.452 at the 3 h time point. Transcript levels of CCR1 and Cxcl2 are upregulated at both time points in the present study and these genes are associated with the recruitment and accumulation of helper T lymphocytes at the cutaneous site of TBEV-infected tick feeding (Figure 4). Selectin L (SELL) is a cellular adhesion molecule found on the surface of most lymphocytes that mediates lymphocyte capture and rolling at sites of inflammation (Tedder et al., 1995). In the present study, SELL transcription was upregulated at the 1 h time point, contributing to the IPA prediction that helper T lymphocytes are accumulating, but the downregulated transcription of SELL at 3 h is inconsistent with the predicted activation pattern, as depicted by the yellow arrow in Figure 4. TLR9, a receptor that preferentially binds unmethylated CpG sequences in bacterial and viral DNA, was downregulated in the present study (Figure 4). Here, the downregulation of TLR9 transcription is predicted to activate the accumulation of T lymphocytes at the skin feeding site of a TBEV-infected tick. This prediction is supported by a recently developed mouse model of cutaneous lupus that is dependent on the expression of TLR7 and the loss of TLR9, which ultimately results in extensive accumulation of IFNγ-producing T lymphocytes (Mande et al., 2017). Distal-less 3 (DLX3) is a transcription factor involved in the terminal differentiation of keratinocytes. In a mouse model with epidermal ablation of DLX3, there was an increase of IL17-producing CD4^+^ T cells, CD8^+^ T cells, and γδ T cells in the skin and draining lymph nodes (Hwang et al., 2011); thus, the downregulated transcription of DLX3 in the skin biopsies from the present study likely contributes to the accumulation of helper T lymphocytes at the cutaneous feeding site of infected ticks (Figure 4).

“Maintenance of cells” was the fourth predicted bio-function in the IPA core comparison analysis. This bio-function was predicted to be inhibited at both experimental time points (1 h activation z-score = −1.342, 3 h activation z-score = −2.236). The “maintenance of cells” activation network is very similar to the top predicted network, “maintenance of leukocytes”; however, the only difference is that Sonic hedgehog (SHH) is present in the “maintenance of cells” network (Figure 4). The touch dome, a highly specialized mechanosensory epidermal structure composed of distinct keratinocytes in close association with innervated Merkel cells, is maintained by signaling with SHH to tissue-specific stem cells (Xiao et al., 2015). In the present study SHH transcription is upregulated after 1 h of TBEV-infected tick feeding, a finding that is inconsistent with the predicted pattern of inhibited cell maintenance (depicted by the yellow arrow in Figure 4); however, at the 3 h time point, SHH transcription was downregulated, contributing to the predicted inhibition of touch dome cell maintenance (Figure 4). Some damage to the perineural skin microenvironment is likely to occur because of the mechanical injury induced by tick feeding, and this could ultimately impact SHH signaling and the touch dome homeostasis.

The “activation of neutrophils” was the fifth predicted bio-function in the core comparison analysis. After 1 h of TBEV-infected vs. uninfected tick feeding, neutrophil activation was predicted to be activated (activation z-score = 1.342); however, after 3 h, neutrophils were predicted to be inhibited (activation z-score = −2.236) (Figure 4). The upregulated transcription of Cxcl5 and TREML2 at the 1 h time point, followed by the downregulation of these genes at the 3 h time point contribute to this phenomenon because Cxcl5 is a chemokine involved in the activation of neutrophils (Proost et al., 1993) and TREML2 potentiates neutrophil activation in response to G protein-coupled receptor signaling (Halpert et al., 2011). Likewise, Lipocalin-2 (LCN2) transcription was upregulated at 1 h post-TBEV-infected tick feeding and downregulated at 3 h. LCN2 is expressed in neutrophils and is a potent inducer of their chemotaxis and migration to sites of inflammation (Schroll et al., 2012); thus, its upregulation at the 1 h time point further contributes to the activation of neutrophils at the feeding site of TBEV-infected ticks. VTCN1 belongs to the B7 family of costimulatory proteins. VTCN1 inhibits the expansion of neutrophils from their progenitors, as demonstrated by VTCN1-deficient mice that display enhanced neutrophil-mediated innate immunity (Zhu et al., 2009); therefore, the downregulation of VTCN1 transcription after 1 h, and its upregulation after 3 h, contributes to the activation of neutrophils at the cutaneous feeding site of a TBEV-infected tick (Figure 4).

### Immunohistochemistry

**Figure 5:**
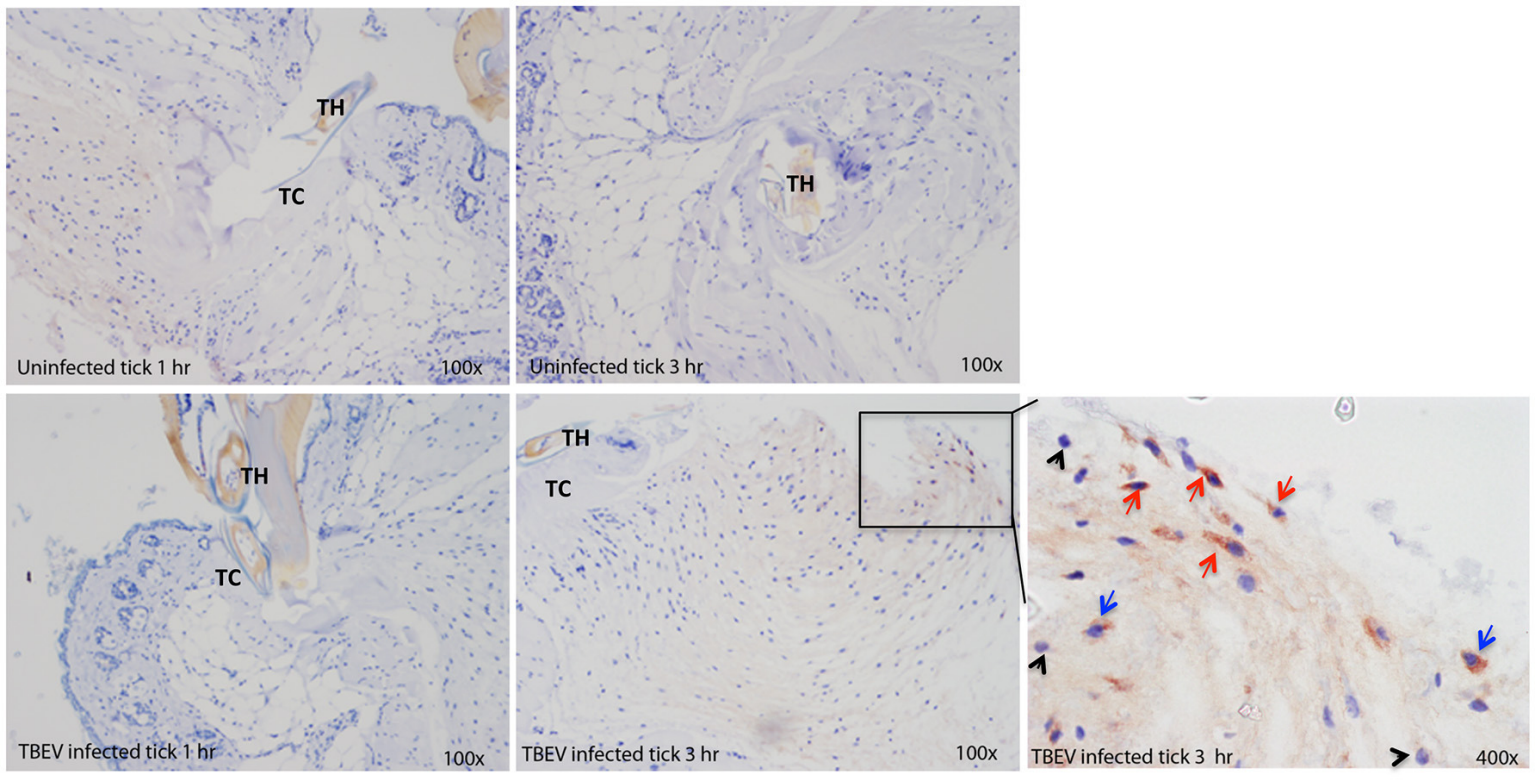
Immunohistochemistry of the *Ixodes ricinus* feeding loci. Five micron sections from skin biopsies harvested at *I. ricinus* feeding sites were subjected to immunohistochemistry procedures to detect TBEV antigens. Red arrows point to TBEV infected fibroblasts; blue arrows point to TBEV infected mononuclear phagocytes, and black arrowheads point to uninfected neutrophils. TH, tick hypostome; TC, tick cement.

Immunohistochemical analysis was performed at the feeding sites of TBEV-infected and uninfected *I. ricinus* to determine if the gene expression data could be correlated to the tissue morphology and inflammation. The tick feeding site is characterized by extravasated erythrocytes and leucocytes, and with a steady influx of inflammatory cells. As early as 1 h post-TBEV-infected tick feeding, neutrophils were observed at both infected and uninfected tick feeding sites, with a marked increase at the infected tick feeding site. After 3 h of TBEV-infected tick feeding, increased recruitment of inflammatory cells was observed at the infected tick feeding site compared to the uninfected tick feeding site. Among these, TBEV antigens were localized in fibroblasts and mononuclear cells, but not in neutrophils (Figure 5). These immunohistochemistry observations support the cutaneous immune gene expression data, showing an inflammatory micro-environment at the feeding site of TBEV-infected ticks. The recruitment of inflammatory cells appears to be much more pronounced in the TBEV-infected tick feeding site than the uninfected tick feeding site.

## Conclusions

The present study demonstrates that TBEV-infected *I. ricinus* adult ticks create an inflammatory environment at the murine cutaneous interface within 1 h of feeding. Significant differences in immune responses were observed between hosts infested with TBEV-infected vs. uninfected ticks. Furthermore, genes associated with neutrophil activation and mobilization were modulated in the presence of TBEV, suggesting that there is an influx of neutrophils and other phagocytic inflammatory cells to the tick feeding site after only 1 and 3 h of infected tick feeding. Immunohistochemistry further supported the cutaneous immune gene expression analysis, demonstrating pronounced recruitment of inflammatory cells, especially neutrophils, to the feeding site of TBEV-infected ticks. Taken together with our earlier study on POWV-infected tick feeding sites (Hermance and Thangamani, 2014), and the current study, it is clearly evident that during the earliest stages of flavivirus-infected tick feeding, a complex, inflammatory micro-environment is created in the host's skin. The present study serves to expand our understanding of the immunological events that occur at the cutaneous interface during the early stages of tick-borne flavivirus transmission to a host.

## Author contributions

Conceived the idea: ST; Provided reagents and materials: ST and MK; Performed experiments: ST, MK, MH, RS, SW, MS, and DH; Data analysis: ST, MH, and SW; Wrote manuscript: ST and MH. All authors critically read and revised the manuscript.

### Conflict of interest statement

The authors declare that the research was conducted in the absence of any commercial or financial relationships that could be construed as a potential conflict of interest.
