# Supplementary material for: Transcriptional Immunoprofiling at the Tick-Virus-Host Interface during Early Stages of Tick-Borne Encephalitis Virus Transmission
Source: Front Cell Infect Microbiol. 2017 Dec 1;7:494. doi: 10.3389/fcimb.2017.00494 (PMC5716978; doi:10.3389/fcimb.2017.00494)
Supplement: Supplementary file 2 [file DataSheet2.docx]

Supplement Table 1: List of primers used in validation study.

| **Gene name** | **Forward (5’-3’)** | **Reverse (5’-3’)** |
| --- | --- | --- |
| Ccl7 | tttgtttcttgacatagcagcat | tctcactctctttctccacca |
| Ifng | agataatctggctctgcagga | gtcattgaaagcctagaaagtctg |
| Cxcl5 | gatccagacagacctccttct | ttgatcgctaatttggaggtga |
| Myd88 | cccaacgatatcgagtttgt | ttcttcatcgccttgtattt |
| Il1b | cgagatttgaagctggatgc | tgacagtgatgagaatgacctg |
| Nfkb | atggcagacgatgatccctac | cggaatcgaaatcccctctgtt |
| Il2 | gcagaggtccaagttcatcttc | gcaggatggagaattacaggaa |
| Stat3 | agctcctcagtcacgatca | gttcaagcacctgacccttag |
| Il4 | tgatgctctttaggctttccag | cagagactctttcgggcttt |
| Tlr3 | ctcacctccaca tcttgaa t | ctgttcaggttttgaaaagg |
| Il6 | gcaagtgcatcatcgttgttc | agtcggaggcttaattacacat |
| Tnfa | tctttgagatccatgccgttg | agaccctcacactcagatca |
| Il10 | atggccttgtagacaccttg | gtcatcgatttctcccctgtg |
| Il12a | ctctcgttcttgtgtagttcca | acagatgacatggtgaagacg |
| Gapdh | gtggagtcatactggaacatgtag | aatggtgaaggtcggtgtg |
